# The validity and reliability of Indonesian version of atrial fibrillation effect on quality of life (AFEQT) questionnaire for atrial fibrillation patients

**DOI:** 10.1186/s41687-023-00672-x

**Published:** 2023-12-15

**Authors:** Putri Zulmiyusrini, Muhammad Yamin, Muhadi Muhadi, Juferdy Kurniawan, Simon Salim

**Affiliations:** 1 Department of Internal Medicine, dr. Cipto Mangunkusumo National General Hospital, Faculty of Medicine, Universitas Indonesia, Jakarta, Indonesia; 2 Division of Cardiology, Department of Internal Medicine, Faculty of Medicine, National General Hospital, Universitas Indonesia, Jakarta, Indonesia; 3 Division of Hepatobiliary, Department of Internal Medicine, Faculty of Medicine, National General Hospital, Universitas Indonesia, Jakarta, Indonesia

**Keywords:** Atrial fibrillation, Quality of life, AFEQT, Questionnaire, Validation

## Abstract

**Background:**

More than 60% of patients with atrial fibrillation (AF) have a significant health-related quality of life (HRQoL) impairment. HRQoL, a patient-reported outcome (PRO), has become an important endpoint to assess treatment success in AF patients. The Atrial Fibrillation Effect on Quality of Life (AFEQT) questionnaire is an AF-specific HRQoL tool shown to be feasible, reliable, and valid, with translations in various languages. Since this questionnaire has never been translated or validated in Indonesian, we aimed to determine the validity and reliability of the Indonesian version of the AFEQT questionnaire for AF patients.

**Results:**

This cross-sectional, observational study was conducted in the Integrated Cardiovascular Service Polyclinic, Cipto Mangunkusumo Hospital, Indonesia, from December 2021 to March 2022. A total of 30 participants were recruited for cross-cultural adaptation process, which consisted of translation and adaptation process, and a total of 102 participants were consecutively recruited to participate in the validation process, which consisted of validity test (construct validity) and reliability tests (internal consistency and test-retest). The retest was conducted within a 1–2-week interval after the baseline assessment, by analyzing the intraclass correlation coefficient (ICC). The construct validity was determined by multitrait scaling analysis, and the convergent and divergent validity was compared to SF-36 domains. Multitrait scaling analysis revealed that all items in the Indonesian version of the AFEQT questionnaire had a strong negative correlation towards their respective domains (r -0.639–-0.960). For convergent and divergent validity, AFEQT domains had weak to strong positive correlations to all SF-36 domains (r 0.338–0.693). This questionnaire also had acceptable internal consistency (Cronbach’s α for overall score: 0.947; Domains: Symptoms: 0.818, Daily Activities: 0.943, Treatment Concern: 0.894, and Treatment Satisfaction: 0.865), as well as moderate-to-good test-retest reliability (0.521–0.828).

**Conclusions:**

The Indonesian version of the AFEQT questionnaire has good validity and reliability for assessing quality of life of atrial fibrillation patients in Indonesia.

## Introduction

Atrial fibrillation (AF) is the most common arrhythmia in adults and the leading cause of stroke, heart failure, sudden death, and cardiovascular morbidity worldwide. AF prevalence is increasing globally, with current estimates at 2–4% in adults. Greater prevalence is found in older persons and patients with other comorbidities, such as hypertension, diabetes mellitus, heart failure (HF), coronary artery disease (CAD), chronic kidney disease (CKD), obesity, and obstructive sleep apnea (OSA) [1]. The prevalence rate in Southeast Asia shows a similar trend, such as Singapore at 1.5%, Thailand at 0.4–2.2%, and Malaysia at 0.5–0.7%, which are relatively lower than that of other countries [2]. In Indonesia, no recent epidemiological study of AF has been performed nationally, to the best of our knowledge. A multinational observational study, the Monitoring of Trend and Determinant in Cardiovascular Disease (MONICA), was conducted on the urban population in Jakarta in 1998 and found that the incidence of AF was 0.2%, with a male-to-female ratio of 3:2 [3]. A more recent study conducted in Makassar in 2014–2018 reported an AF prevalence of 0.96% [4].

Patients with AF are at risk of stroke and experience various symptoms, such as lethargy, palpitations, dyspnea, and chest tightness, all of which decrease health-related quality of life (HRQoL) and place a significant burden on patients, public health systems, and the health economy [1, 5]. More than 60% of AF patients have a significant quality of life (QoL) impairment. QoL is significantly lower in women, young individuals, and patients with comorbidities. The reduction of QoL is associated with a high risk of hospitalization [6]. Therefore, QoL is an important cardiovascular health outcome in clinical practice.

QoL is subjective and defined as a perception of discrepancy between actual and desired functional status and the overall impact of disease on the well-being of a given patient [7]. QoL is a type of Patient-Reported Outcome (PRO) and a subject of interest as an endpoint in clinical trials to improve care and assess treatment success from the patient’s perspective [5]. QoL can now be quantified using a validated general and disease-specific assessment tool. More than 10 AF-specific QoL tools have been validated to assess QoL in patients with AF. Compared to other AF-specific instruments, the Atrial Fibrillation Effect on Quality of Life (AFEQT) questionnaire has the best psychometric properties. It explicitly measures patients’ perceptions of their symptoms, functional impairment, treatment concerns, and satisfaction with treatment [9]. It has been used in various QoL studies in AF patients and has been translated and validated into 24 languages, including Chinese, Greek, Russian, and Turkish [10–14]. Translations of the AFEQT into other languages showed highly comparable psychometric properties to the original version of the AFEQT, which was used in the US and Canada [9]. Indonesia, the fourth largest country in the world and with increasing life expectancy, has been noted for its increasing AF prevalence. To date, there is no Indonesian version of any AF-specific QoL questionnaire. Therefore, a culturally and linguistically relevant instrument to assess QoL for AF patients in Indonesia is essential.

To address the need for a comprehensive measure of self-reported AF outcomes, a translation and cultural adaptation process must be conducted. A Translation and Cultural Adaptation (TCA) group was formed by The Professional Society for Health Economics and Outcomes Research (ISPOR) in 1999 to create guidelines for the translation and cultural adaptation of PROs. The group reviewed several guidelines available for translation and cultural adaptation and summarized the translation process into ten steps (preparation, forward translation, reconciliation, back translation, back translation review, harmonization, cognitive debriefing, review of cognitive debriefing results and finalization, proofreading, and final report) [15]. However, in this study, we used the cross-cultural adaptation guideline from Beaton and Guillemin, which consists of 6 stages (translation, synthesis, back translation, expert committee review, test of the prefinal version, and submission of documentation to the committee) to translate and validate the AFEQT questionnaire among Indonesian AF patients, as our team was more familiar with and had previously used it for translating the SF-36 and AQUAREL questionnaires into the Indonesian language [16–19].

## Methods

### Setting and study design

This cross-sectional, observational study with consecutive sampling and a survey method of data collection was conducted in the Integrated Cardiovascular Service Polyclinic in Cipto Mangunkusumo National Referral Hospital, Jakarta, Indonesia, over a period of 4 months from December 2021 to March 2022.

### Study population

Eligible subjects were Indonesian-speaking outpatients, 18 years or older, with documented paroxysmal, persistent, longstanding persistent, or permanent AF who were willing to participate in this study. Patients were invited to participate at the time of their scheduled clinic or procedural visit, and those who joined provided written informed consent. Patients with cognitive impairment or active psychiatric illnesses, physical disabilities (handicaps) that interfered with their ability to fill the questionnaire, as well as those treated or hospitalized for severe acute or chronic conditions or who underwent cardiac surgery within 30 days of the recruitment time were excluded from the study. A total of 30 participants were recruited for cross-cultural adaptation, based on minimal requirement sample in the guidelines by Beaton et al. [16]. A total of 102 participants were consecutively recruited for the validity study based on minimum of 100 participants required to fulfil a ratio of 5 participants to each item of the AFEQT questionnaire.

### Study instruments

Atrial Fibrillation Effect on Quality of Life (AFEQT) Questionnaire.

The 20-item AFEQT questionnaire was developed and validated by Spertus et al. [9] to assess the impact of AF and its treatment on patient symptoms, functioning, and daily activities through the following four domains: Symptoms (4 items), Daily Activities (8 items), Treatment Concern (6 items), and Treatment Satisfaction (2 items) [9]. It was developed as a self-administered instrument, using a 4-week recall frame, with all items rated on a 7-point Likert scale. The Likert responses ranged from no limitations/symptoms [1] to the most severe limitations/symptoms [7] in each domain. Only three domains (Symptoms, Daily Activities, and Treatment Concerns) were included in the overall score calculation, indicating the patient’s quality of life. The two items in the Treatment Satisfaction domain were not included in the overall score, as they did not assess patients’ health status. The raw score of 1 to 7 in the overall and domain scores were transformed to a scale of 0 to 100. Higher scores indicate better health status or QoL overall and for each domain. The scoring manual was provided in the supplementary material. We obtained permission via licensing from SJM/Abbott to use the questionnaire and its content.

### SF-36

The 36-item Short Form Health Survey is well-validated, reliable, and the most commonly used general health status measure. It has also been widely used in various studies to assess the AF patients’ quality of life [20–25]. Many studies have addressed the content, construct, concurrent, and predictive value of SF-36. Systematic comparisons indicate that SF-36 includes eight most frequently represented health concepts. Reliability statistics have exceeded the minimum standard of 0.70 in group comparison. The SF-36 questionnaire consists of 8 domains: Physical Functioning (PF), Role-Physical (RP), Bodily Pain (BP), General Health (GH), Vitality (VT), Social Functioning (SF), Role-Emotional (RE), and Mental Health (MH) [26]. Mental (MCS) and Physical (PCS) Component Summary scores are generated to make it possible to reduce the number of statistical comparisons (from eight to two) in analyzing between the SF-36 physical and mental health outcomes. A four-week recall period with a variety response scale is used, with higher scores indicating better health status. Since the weights vary among items, the score item should be recoded into a range of 0–100, where 0 represents the lowest score and 100 represents the highest. The scoring instruction can be accessed from the RAND website [27]. The Indonesian Version of SF-36 has been validated in pacemaker patients and shown to have acceptable internal consistency and test-retest reliability [18].

### Study procedures

This study followed the cross-cultural adaptation guidelines by Beaton et al., which consists of 6 stages (translation, synthesis, back translation, expert committee review, test of the prefinal version, and submission of documentation to the committee) [16]. We divided the procedure into two main processes, translation and cultural adaptation.

### Translation process

Two professional native Indonesian translators independently performed the forward translation from the original English version to the Indonesian language. With input from the research team, the translators reconciled differences between translations to reach a consensus and develop one synthesized Indonesian version of the AFEQT. Two independent native English translators then performed the back-translation from the Indonesian version to English. The research team then compared the back-translated version to the original English version to identify any translation errors. The translation procedures were documented by the authors.

### Cultural adaptation process

The prefinal version of the Indonesian AFEQT was completed by 30 patients with documented AF. The patients were asked to complete the questionnaire and were interviewed to get their opinions about the items and responses in the questionnaire. The results were discussed among the researchers who finalized the questionnaire.

### Statistical analysis

Construct validity is one of the minimum standards for PRO measurements [28]. We used a multitrait scaling analysis approach to analyze for correlations between the item and its domain. The correlation between an item and its domain is expected to be higher than the correlation between an item and other domains. For convergent and divergent validity, we used the Indonesian version of SF-36 as a comparison, since there are no other Indonesian versions of AF-specific questionnaires available at the moment. Such correlations are considered to be weak for correlation coefficients of 0.1–0.39, moderate for 0.40–0.69, strong for 0.70–0.89, and very strong for 0.90–1.00 [29]. Spearman’s correlation coefficient was used to analyze the AFEQT (Indonesian version) total and domain scores and each SF-36 (Indonesian version) domain and summary measure scores.

Internal consistency and test-retest reliability were assessed to fulfill the minimum standard needed to assess the reliability of a PRO measure. Internal consistency, measured by Cronbach’s α, refers to how consistent the items within a scale are and how each item measures aspects of the same underlying domain. Reports have differed on acceptable Cronbach’s α values, ranging from 0.7 to 0.95 [30]. The α value is influenced by the lengths of the scale and the sample size [31]. Therefore, we considered a value of 0.70 or higher to be acceptable or ideal [28, 30, 32]. Test-retest reliability was used to evaluate the stability of the responses over time (at 1-2-week intervals) by assessing the intraclass correlation coefficient (ICC). A value of 0.5–0.75 was considered to be moderate, 0.75–0.9 was considered to be good, and higher than 0.9 was considered to be excellent stability [33]. All analyses were performed using SPSS version 22.0 (IBM) with a two-sided level of significance of 1%.

## Results

### Sociodemographic and medical characteristics of participants

**Fig. 1:**
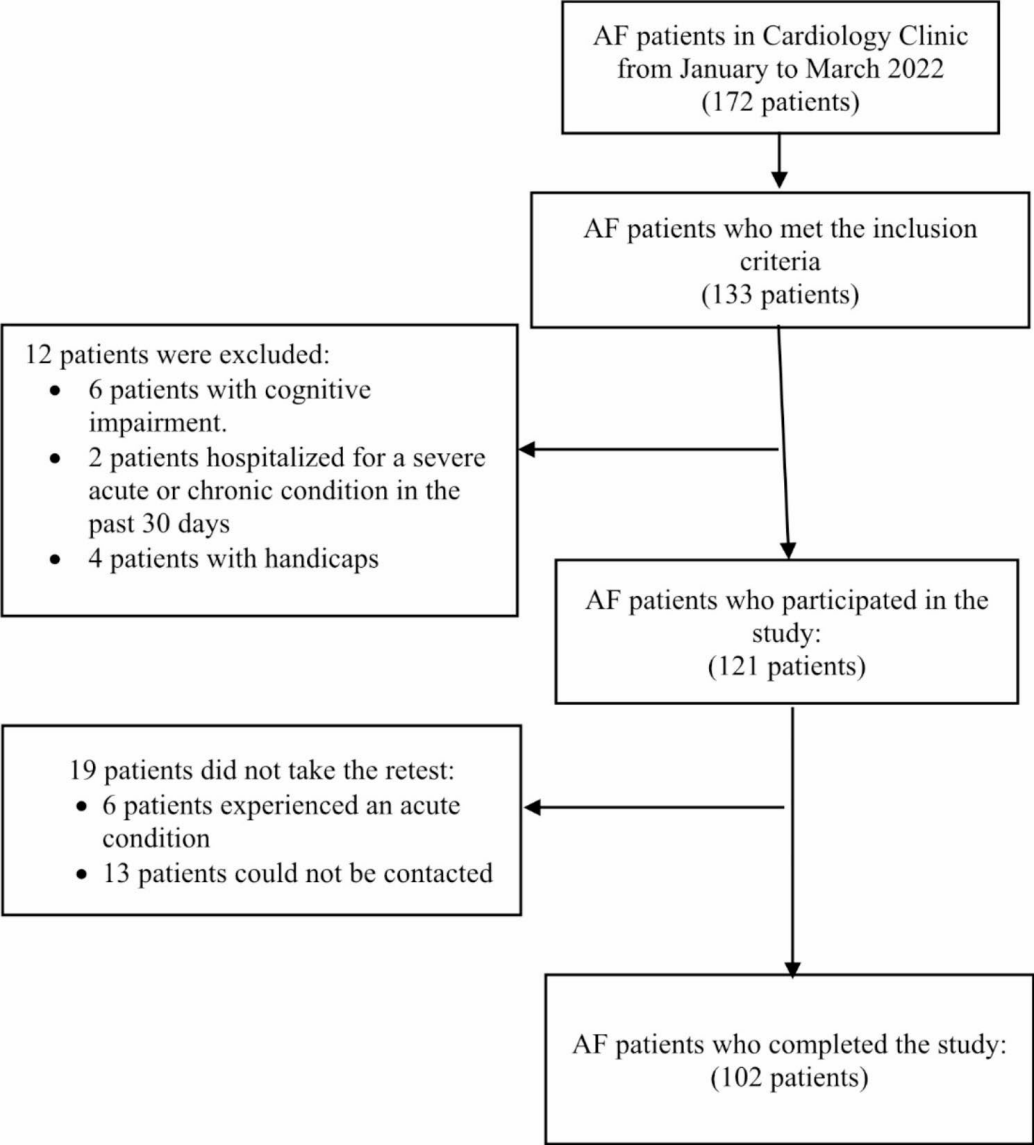
Flow diagram of patient selection

A total of 172 AF patients visited the Cardiology Clinic from January to March 2022, of whom 133 patients met the inclusion criteria. Twelve patients were excluded due to cognitive impairment, recent hospitalization, or physical disabilities. Of 121 patients who consented to participate, 6 were excluded because of acute conditions and 13 others could not be contacted for the retest procedure, leaving 102 patients who were included in the analyses (Fig. 1). Patients who were excluded did not differ statistically from the included patients. The mean age of participants was 58.98 ± 11.82 years. Overall, most respondents were male (52%), married (82.4%), middle school graduates (58.8%), and unemployed (71.6%). Approximately half of the participants had permanent AF (51%) and were classified as EHRA (European Heart Rhythm Association) 2a (51%). Subjects’ median CHA_2_DS_2_-VASc [congestive heart failure, hypertension, age ≥ 75, diabetes, stroke, vascular disease, age 65 to 74 and sex category (female)] score was 3, indicating a high stroke risk among participants, and 95.1% of participants received vitamin K anticoagulants (VKA). The sociodemographic and medical characteristics of the 102 participating AF patients are presented in Table 1. Patients took an average of 10.04 min (range 1.55 to 27.57 min; median 9.46 min) to complete the 20-item Indonesian version of the AFEQT. Around 70% of patients took more than 15 min to fill out the questionnaire and most of them were over 70 years of age.

**Table 1:** Sociodemographic and medical characteristics of 102 participating AF patients

Demographics	(n = 102)
Age, years, mean (SD)	58.98 (11.82)
Sex, n (%)	
Male	53 (52)
Female	49 (48)
BMI, median (IQR)	25.2 (6.09)
Marital status, n (%)	
Married	84 (82.4)
Single	5 (4.9)
Widowed	13 (12.7)
Education, n (%)	
Elementary School	9 (8.8)
Middle School	60 (58.8)
Diploma	10 (9.8)
Bachelor or graduate school	23 (22.6)
Employment status, n (%)	
Government employee	3 (2.9)
Private employee	5 (4.9)
Entrepreneur	10 (9.8)
Other	11 (10.8)
Unemployed	73 (71.6)
Type of AF, n (%)	
Paroxysmal	28 (27.4)
Persistent	16 (15.7)
Longstanding persistent	6 (5.9)
Permanent	52 (51)
EHRA class, n (%)	
1	3 (2.9)
2a	52 (51)
2b	30 (29.4)
3	17 (16.7)
CHA_2_DS_2_-VASc score, median (IQR)	3 (2)
Ejection fraction, median (IQR)	58.35 (16.42)
Medication history, n (%)	
Rate control only	93 (91.2)
Rhythm control only	2 (1.9)
Rate and rhythm control	5 (4.9)
VKA anticoagulants	97 (95.1)

### Translation and cultural adaptation process

Item number 3 in the prefinal questionnaire led to uncertainty among 26% of patients because they actually did not have symptoms of a pause in heart activity. The research team decided not to make any revisions because the choice of words was considered to be appropriate, and was kept through the validation step to evaluate the psychometric properties of the item and the whole questionnaire.

### Construct validity

#### Multitrait scaling analysis

All the items in the questionnaire had strong negative correlations to their own domain, and the correlation was higher compared to other domains in which the items were not assigned. A negative correlation was obtained because a scale of 1 on the questionnaire indicated no limitations/symptoms and a scale of 7 indicated the most severe limitations/symptoms, hence, the higher the scale chosen, the worse the patient’s quality of life. Item number 3 had the weakest correlation in its own domain and overall score. The Multitrait Scaling Analysis is presented in Table 2.

**Table 2:** The Multitrait Scaling Analysis Approach

	Domains				
AFEQT Items	Symptoms*	Daily Activities*	Treatment Concern*	Treatment Satisfaction*	Overall Score*
Symptoms					
1	-0.818	-0.512	-0.543	-0.410	-0.652
2	-0.858	-0.629	-0.597	-0.489	-0.740
3	-0.639	-0.413	-0.470	-0.392	-0.526
4	-0.749	-0.570	-0.454	-0.354	-0.652
Daily Activities					
5	-0.649	-0.820	-0.647	-0.333	-0.828
6	-0.594	-0.727	-0.572	-0.334	-0.734
7	-0.630	-0.873	-0.553	-0.440	-0.823
8	-0.671	-0.829	-0.490	-0.481	-0.794
9	-0.594	-0.843	-0.551	-0.372	-0.797
10	-0.607	-0.869	-0.494	-0.519	-0.796
11	-0.584	-0.869	-0.430	-0.498	-0.765
12	-0.481	-0.820	-0.356	-0.435	-0.678
Treatment Concern					
13	-0.686	-0.529	-0.780	-0.398	-0.712
14	-0.622	-0.469	-0.808	-0.327	-0.666
15	-0.526	-0.514	-0.807	-0.387	-0.696
16	-0.468	-0.392	-0.841	-0.394	-0.618
17	-0.456	-0.432	-0.788	-0.351	-0.639
18	-0.495	-0.412	-0.813	-0.462	-0.623
Treatment Satisfaction					
19	-0.452	-0.460	-0.413	-0.914	-0.502
20	-0.561	-0.498	-0.439	-0.960	-0.551

*Correlation is significant at the 0.01 level (two-tailed). Bolded numbers indicate number of items allocated to each domain. The Treatment Satisfaction domain was not included in Overall Score analysis

#### Convergent and divergent validity

All the domains in the Indonesian AFEQT questionnaire had weak to strong positive correlations with the Indonesian version of the SF-36 questionnaire. The AFEQT Daily Activities domain had stronger correlations to SF-36 Physical Functioning, Role-Physical, Vitality domains, and PCS score compared to the SF-36 Social Functioning, Role-Emotional, Mental Health, General Health domains, and MCS score. Conversely, the AFEQT Treatment Concern domain had weaker correlations to SF-36 Physical Functioning and Role-Physical domains and PCS score, compared to the SF-36 Role-Emotional and Mental Health domains as well as MCS score. The AFEQT Symptoms domain had moderate correlations to most of the domains in SF-36, except for the Physical Functioning domain which showed a weak correlation. On the other hand, AFEQT Treatment Satisfaction domain had weak correlations to most SF-36 domains. AFEQT overall score had strong correlations to both PCS and MCS scores. The correlation between the Indonesian Version of AFEQT and SF-36 domains are presented in Table 3.

**Table 3:** Correlation between the Indonesian version of AFEQT and SF-36 domains

AFEQT Domains	SF-36									
	Domains					Components				
	PF*	RP*	BP*	GH*	VT*	SF*	RE*	MH*	PCS*	MCS*
Symptoms	0.372	0.528	0.503	0.498	0.561	0.443	0.466	0.570	0.548	0.621
Daily Activities	0.617	0.677	0.603	0.524	0.693	0.415	0.517	0.561	0.746	0.681
Treatment Concern	0.430	0.483	0.372	0.480	0.532	0.493	0.494	0.569	0.539	0.649
*Treatment Satisfaction*	*0.338*	*0.387*	*0.358*	*0.521*	*0.455*	*0.345*	*0.338*	*0.386*	*0.453*	*0.467*
Overall Score	0.586	0.667	0.574	0.558	0.693	0.498	0.574	0.634	0.730	0.746

*Correlation is significant at the 0.01 level (two-tailed). Bolded numbers indicate correlations in the AFEQT and SF-36 domains which have similar types of questions

The Treatment Satisfaction domain was not included in Overall Score analysis

#### Reliability

Cronbach’s α was 0.947 for the overall Indonesian version of AFEQT and as follows for the four domains: Symptoms (0.818), Daily Activities (0.943), Treatment Concern (0.894), and Treatment Satisfaction (0.865). All these values indicated acceptable or ideal internal consistency [30, 32]. The ICCs, indicators of test-retest reliability, were considered good for overall score (0.862), Symptoms (0.791), Daily Activities (0.842), and Treatment Concern (0.824), and moderate for the Treatment Satisfaction domain (0.715). However, at the item level, only 50% of items had ICC > 0.75, while the others had ICC 0.5–0.75. The reliability analysis is presented in Tables 4 and 5.

**Table 4:** The ICC of items in the Indonesian version of AFEQT

Item	ICC (95%CI)
Symptoms	
1	0.790 (0.687–0.858)
2	0.742 (0.613–0.828)
3	0.604 (0.414–0.732)
4	0.719 (0.585–0.810)
Daily Activities	
5	0.668 (0.509–0.776)
6	0.681 (0.527–0.784)
7	0.760 (0.646–0.838)
8	0.767 (0.656–0.843)
9	0.774 (0.667–0.847)
10	0.776 (0.668–0.849)
11	0.815 (0.726–0.875)
12	0.763 (0.648–0.840)
Treatment Concern	
13	0.788 (0.687–0.857)
14	0.828 (0.744–0.884)
15	0.715 (0.579–0.807)
16	0.724 (0.592–0.813)
17	0.781 (0.643–0.861)
18	0.640 (0467-0.757)
Treatment Satisfaction	
19	0.521 (0.296–0.675)
20	0.734 (0.607–0.820)

The Treatment Satisfaction domain was not included in Overall Score analysis

**Table 5:** The cronbach’s α coefficient and ICC of the Indonesian version of the AFEQTTable_caption

AFEQT Domains	Cronbach’s α	ICC (95%CI)
Symptoms	0.818	0.791 (0.683–0.861)
Daily Activities	0.943	0.842 (0.766–0.893)
Treatment Concern	0.894	0.824 (0.733–0.884)
Treatment Satisfaction	0.865	0.715 (0.578–0.807)
Overall Score	0.947	0.862 (0.792–0.908)

## Discussion

Quantifying patients’ perceptions by measuring their HRQoL is an important measure to evaluate treatment success and improve patient care [1]. Patients with AF mostly have impaired QoL, independent of other cardiovascular conditions, because they experience a variety of symptoms [5]. Compared to other AF-specific instruments, AFEQT is more comprehensive in capturing the disease and its management impact on patients’ physical and emotional function. It has also been used in many large-scale clinical and observational studies on the quality of life in AF patients [6, 23, 34–36]. However, before the original English version can be used in non-English-speaking populations, the questionnaire must undergo translation as well as cultural adaptation and validation to ensure that it has the same objective, properties, and function as the original questionnaire. We translated the AFEQT into the Indonesian language and established its psychometric properties in Indonesian AF patients.

For the translation and cultural adaptation processes, we used a guideline developed by Beaton et al. This guideline has a similar outline to the one published by the ISPOR task force, which consists of forward translation, synthesis into one forward translation (reconciliation), back translation, expert committee review (back translation review), and test of the prefinal version in a small group of relevant patients (cognitive debriefing) [16]. We did not perform harmonization to compare the back translation of multiple language versions with each other due to lack of data of the other language versions.

The baseline characteristics of participants in terms of educational background and employment status were quite different from the original study in the US, in which the majority of participants were full-time or part-time workers and had college educational backgrounds. These differences might have affected patients’ understanding of the questionnaire and raised the question of whether this questionnaire was applicable to populations with different academic backgrounds and employment statuses. However, the characteristics of gender, marital status, educational background, and employment status in our study were somewhat similar to the adaptation studies in Turkey and China, in which most participants had at least secondary education and were unemployed [11, 12]. Thus, a translated AFEQT questionnaire could also be implemented in Indonesia. Moreover, the results showed that the Indonesian version of AFEQT was highly comparable with psychometric properties reported in the original version and adapted versions, such as those in Russian, Greek, Turkish, and Chinese [9, 11–14].

Content validity, construct validity, and responsiveness are considered to be the minimum standards for a PRO measure [28]. However, since our study objective was to validate the adapted version of the established AFEQT, we did not assess the content validity. Responsiveness was not assessed since that would need a longitudinal study. Therefore, we assessed construct validity in our study (multitrait scaling analysis, convergent and divergent validity) to measure the expected association against other instruments which measure the same construct [28].

Our results showed that the Indonesian version of AFEQT had an appropriate construct validity by demonstrating an adequate multitrait scaling analysis as well as convergent and divergent correlations to the Indonesian version of SF-36. In multitrait scaling analysis, all the items had higher correlations to the domain they were assigned to compared to other domains. Item number 3 in the Indonesian version of AFEQT had the lowest correlation, both to its domain and overall score. 26% of participants in the prefinal testing stage exhibited poor understanding of the question. This finding was also supported by a Chinese study in which item 3 had the lowest factor loading compared to other items [12] Therefore, item 3 is a cause of concern for the future use of the questionnaire. For convergent and divergent validity, the overall score of the Indonesian AFEQT showed a moderate-to-strong correlation with all domains in the Indonesian version of SF-36. The AFEQT Daily Activities domain correlated well with the physical component (PCS) of SF-36, while the Treatment Concern domain correlated better with the mental component (MCS) of SF-36 compared to the physical component. These results were similar to the original AFEQT study by Spertus et al. [9]. However, the Symptom domain had a weak correlation to the Physical Functioning domain in SF-36. Patients’ impaired functional status is not solely due to AF symptoms and is more influenced by cardiovascular conditions and other risk factors for AF, such as age and gender [37]. Moreover, permanent AF patients predominated our sample; they tend to have a lower symptom burden than patients with other types of AF. On the other hand, the Treatment Satisfaction domain showed a weak correlation to almost all domains in SF-36. This finding was predictable because two satisfaction items do not assess patient health status and were not included in the overall score. Satisfaction items did not have a potential criterion standard to which they could be compared [9]. However, evaluating patient treatment satisfaction is also one of the main characteristics of patient-reported outcomes [38].

In terms of reliability, the Indonesian version of the AFEQT was also shown to be an internally reliable instrument with satisfactory stability, as indicated by the very high Cronbach’s α and ICC for overall score (0.947 and 0.862, respectively). These findings were similar to AFEQT validity studies in Russia, Greece, Turkey, and China, which also had satisfactory questionnaire reliability [11–14]. However, for the ICC, we used a value of 0.75 or greater to indicate good test-retest reliability. This cut-off value was higher than that used in other AFEQT validation studies. Therefore, one domain (Treatment Satisfaction) and half of the items did not meet the criteria of good test-retest reliability. The lowest ICC value was in item number 19 (0.521), followed by item numbers 3 (0.604) and 18 (0.640). This slightly lower value may be attributable to the nature of AF patients who have a quite wide range of symptoms, from asymptomatic and unaware of any treatment for their AF to some debilitating symptoms. The low degree of measurement agreement also contributed to the low ICC [33]. However, ICC analysis at the domain level indicated good test-retest reliability.

This study had several limitations. First, we did not reassess the content validity of the questionnaire. Second, this study was a single-center study with a relatively small sample size compared to the original AFEQT validation study. Third, our sample did not adequately represent AF type, as more than 50% of our subjects had permanent AF. However, this characteristic was not expected to influence the psychometric properties of the questionnaire. Fourth, we did not evaluate the responsiveness of the tool in terms of the ability to detect clinically significant changes in patient health status over time. Further research is needed to evaluate the responsiveness of the Indonesian version in different health settings. Finally, due to the absence of other AF-specific validated questionnaires, we used a generic instrument (SF-36) for convergent and divergent validation, which may not have been able to capture AF-specific symptoms.

## Conclusions

The Indonesian version of the AFEQT was deemed valid, reliable, and comparable to the original English and the other translated versions. Use of the Indonesian AFEQT in clinical settings could further enable health professionals to evaluate patient QOL with regards to AF and treatment, as well as to gain a thorough understanding of how to best give patient-centered care. However, a multicenter study with a longer observation is needed to assess the responsiveness of the Indonesian version of the AFEQT.

## Data Availability

The dataset used and/or analyzed during the current study are available from the corresponding author on reasonable request.

## Abbreviations

AF: Atrial Fibrillation
AFEQT: Atrial Fibrillation Effect on Quality-of-life
BP: Bodily Pain
CAD: Coronary Artery Disease
CHA_2_DS_2_-VASc: Congestive heart failure, Hypertension, Age ≥ 75, Diabetes, Stroke Vascular disease, Age 65 to 74 and Sex category (female)
CKD: Chronic Kidney Disease
EHRA: European Heart Rhythm Association
GH: General Health
HF: Heart Failure
HRQoL: Health-related Quality of Life
ICC: Intraclass correlation coefficient
MCS: Mental Component Summary
MH: Mental Health
OSA: Obstructive Sleep Apnea
PCS: Physical Component Summary
PF: Physical Functioning
PRO: Patient-Reported Outcomes
QoL: Quality of Life
RE: Role-Emotional
RP: Role-Physical
SF: Social Functioning
SF-36: the 36-item Short Form Survey
SPSS: Statistical Package for Social Sciences
VT: Vitality
